# “Swimming against the current”: Behavioral data of *Betta splendens* during an escape and avoidance task with water flows as the aversive stimulus

**DOI:** 10.1016/j.dib.2019.104260

**Published:** 2019-07-15

**Authors:** Camilo Hurtado-Parrado, César Acevedo-Triana, Joseph Pear

**Affiliations:** a Fundación Universitaria Konrad Lorenz, Bogotá, Colombia; b Department of Psychology, Troy University, AL, United States; c School of Psychology, Universidad Pedagógica y Tecnológica de Colombia, Tunja, Colombia; d Department of Psychology, University of Manitoba, Winnipeg, Canada

**Keywords:** *Betta splendens*, Siamese fighting fish, Avoidance, Escape, Negative reinforcement, Water disturbances, Water flows

## Abstract

This paper describes the behavioral data of an experiment in which water flows (WFs) were first used as replacement of the traditional electric shocks to test free-operant avoidance in *Betta splendens* (Hurtado-Parrado et al. 2019 https://doi.org/10.1016/j.beproc.2018.10.021). WFs with a duration of 10 s each were delivered with 30-s flow-flow (F–F) and response-flow (R–F) intervals in a custom-made shuttle tank. Fish escaped or avoided the WFs by changing compartments. Crossings during the WFs, interrupted the flows, were automatically scored as escape (Esc), and initiated a new R–F interval. Crossings that occurred during R–F or F–F intervals were scored as avoidance responses and also reset the R–F interval. We compared the effect of adding a warning stimulus - curtains of air bubbles - to the last 5 s of the R–F interval; i.e., signaled versus unsignaled avoidance. A unique development of the WFs procedure, and thus the data here described, is that crossings were further differentiated into subcategories; namely, early avoidance (EA) if a crossing occurred during the first 25 s of the R–F interval; late avoidance (LA) if a crossing occurred during the last 5 s of the R–F interval; and Flow-Flow avoidance (FF) if a crossing occurred anytime during the F–F interval. Here we present the data of six bettas across the different phases of the experiment; namely, baseline (BL - no WFs programmed), signaled avoidance (SA – warning stimulus scheduled), and unsignaled avoidance (UA - no warning stimulus scheduled). The dataset available at the Open Science Framework (OSF) repository (http://doi.org/10.17605/OSF.IO/FMHXD (Hurtado-Parrado et al., 2019)) includes for each fish and per 20-min daily session the total number of crossings; frequency of each type of crossing (Esc, EA, LA, FF); total WF frequency and duration, the total time spent in each compartment, and an index of preference for each compartment based on the proportion of time spent in the tank's compartments.

**Table:** Specifications table

Subject area	*Psychology*
More specific subject area	*Animal behavior, experimental analysis of behavior.*
Type of data	*Table (Microsoft EXCEL*^®^*spreadsheet)*
How data was acquired	A custom-made video tracking system [2] was used to trace the movements of the fish.
Data format	Raw data in a spreadsheet (Table)
Experimental factors	Baseline (no water flows or warning stimulus), signaled avoidance (water flows every 10 s, curtains of air bubbles during the last 5 s of the response-flow interval – i.e., warning stimulus), and unsignaled (water flows every 10 s, no warning stimulus) conditions.
Experimental features	We developed an experimental set up for testing free-operant avoidance [3] with *Betta splendens* using water flows (WFs) instead of traditional electric shocks. WFs of 10-s duration were presented with 30-s flow-flow (F–F) and response-flow (R–F) intervals. Fish crossed from one compartment of a shuttle tank to the other to interrupt (escape) or prevent (avoid) the WFs. The behavioral effects of adding a warning stimulus - curtains of air bubbles - to the last 5 s of the response-flow interval were explored; i.e., signaled versus unsignaled avoidance.
Data source location	Winnipeg, Manitoba, Canada.
Data accessibility	Open Science Framework repository (OSF): http://doi.org/10.17605/OSF.IO/FMHXD[4]
Related research article	Hurtado-Parrado, C., Acevedo-Triana, C. & Pear, J. (2019). Aversive control of *Betta splendens* behavior using water disturbances: Effects of signaled and unsignaled free-operant avoidance and escape contingencies. *Behavioural Processes, 158*(1), 18–31. https://doi.org/10.1016/j.beproc.2018.10.021

**Table:** 

**Value of the data** • We present data of the first implementation of a protocol that replaced electric shocks with water flows (WFs) to test escape and free-operant avoidance phenomena in *Betta splendens*. We compared the effects of adding a warning stimulus (curtains of air bubbles) to the last 5 s of the response-flow interval; i.e., signaled versus unsignaled avoidance. • A unique aspect of these data, is that the aversive stimulus (WFs) in the protocol had a nonzero duration – i.e., WFs lasted 10 s unless an escape response interrupted them. This aspect allowed to simultaneously (a) gather data on both escape and avoidance behavior, (b) measure the amount of time that the fish was exposed to the aversive stimuli, and (c) test how these measures were affected by presence or absence of the warning stimulus (signaled versus unsignaled conditions). • Adding to the value of these data is the fact that the behavior of interest - crossings between compartments in a shuttle tank - was measured on the basis of different subcategories; namely, early avoidance (EA) if a crossing was displayed during the initial 25 s of the R–F interval; late avoidance (LA) if a crossing occurred during the last 5 s of the R–F interval; and Flow-Flow avoidance (FF) if a crossing occurred anytime during the F–F interval. • The dataset includes an innovative measurement of the subject's preference for the compartments of the shuttle tank based on the ratio of time allocated to each compartment (i.e., preference index). • These data could be used for further insights into the relationship between the frequency of specific behaviors regulated by aversive events (escape and avoidance) and related spatiotemporal patterns, which to date have received considerably less attention [e.g.,5].

## 1 Data

The data of each of the six subjects is provided in a Microsoft EXCEL^®^ file across separate sheets (C01 – C06). Each line of the dataset provides the following data collected per daily 20-min experimental session: (a) session number and phase (baseline, signaled avoidance, or unsignaled avoidance); (b) total number of crossings, (b) type of crossing (escape, avoidance, early avoidance, or late avoidance), (c) total number of water flows delivered, and how many occurred in each compartment of the shuttle tank, (d) total water flow time in seconds and in percentage, (e) amount of time the fish spent in each compartment in seconds, and (f) index of preference for each compartment (calculated as the ratio of time allocated to the right and the left compartments). Data corresponding to (c), (d), (e) and (f) were not analyzed for the original experiment [1]. The total number of observations per subject ranged between 75 and 90.

For instance, sheet C01in line 44 displays the data collected for subject C01 during the first session of the second unsignaled avoidance condition (UA2). During that 20-min session, fish C01 displayed 32 crossings in total, 26 of them were escape responses and 6 were avoidance responses. Among the avoidance crossings, 4 occurred during the F–F interval, and 2 during the first 25-s of the R–F interval (i.e., early avoidance crossings). Throughout the session, 32 WFs were delivered, which represented a 106.7% of the number of WFs scheduled for a subject that never displayed an escape or avoidance response during the session; i.e., 30 WFs (this counterintuitive effect results from the high number of escape responses, which by interrupting the duration of each WF increase on the long run the number of WFs during the same 20-min session).

Fifteen of the WFs were delivered in the right compartment and 17 in the left compartment. All instances of WFs added to a total of 176 s of WF time (58.7% of the total flow time scheduled for the session if the fish were not have had displayed any escape or avoidance crossing; 300 s). Of the total WF time, 91 s occurred in the right compartment and 85 on the left compartment. Lastly, the video tracking system reported that the fish spent in total 594 s in the right compartment and 588 in the left compartment, which result in a 0.01 right/left ratio of time allocation (i.e., no clear preference for a compartment of the shuttle tank).

## 2 Experimental design, materials, and methods

### 2.1 Subjects

Six male bettas were obtained from a local pet store. They were labeled C01, C02, C03, C04, C05, and C06. Their mean length was 6 cm. Each subject was housed in an individual tank (different than the experimental tank). A 12/12 hr. light/dark cycle (lights on at 8:00 AM) was in effect. The daily experimental sessions took place between 10:00 AM and 3:00 PM. Fish were fed in their home tanks 1 hr. prior to the daily experimental session. Data collection on fish C01, C02, and C04 stopped on day 80, 76 and 82, respectively, due to health issues.

All experimental procedures and animal maintenance were approved by the Fort Garry Campus Animal Care Committee of the University of Manitoba (Protocol No. F12-024).

### 2.2 Instruments

**Fig. 1:**
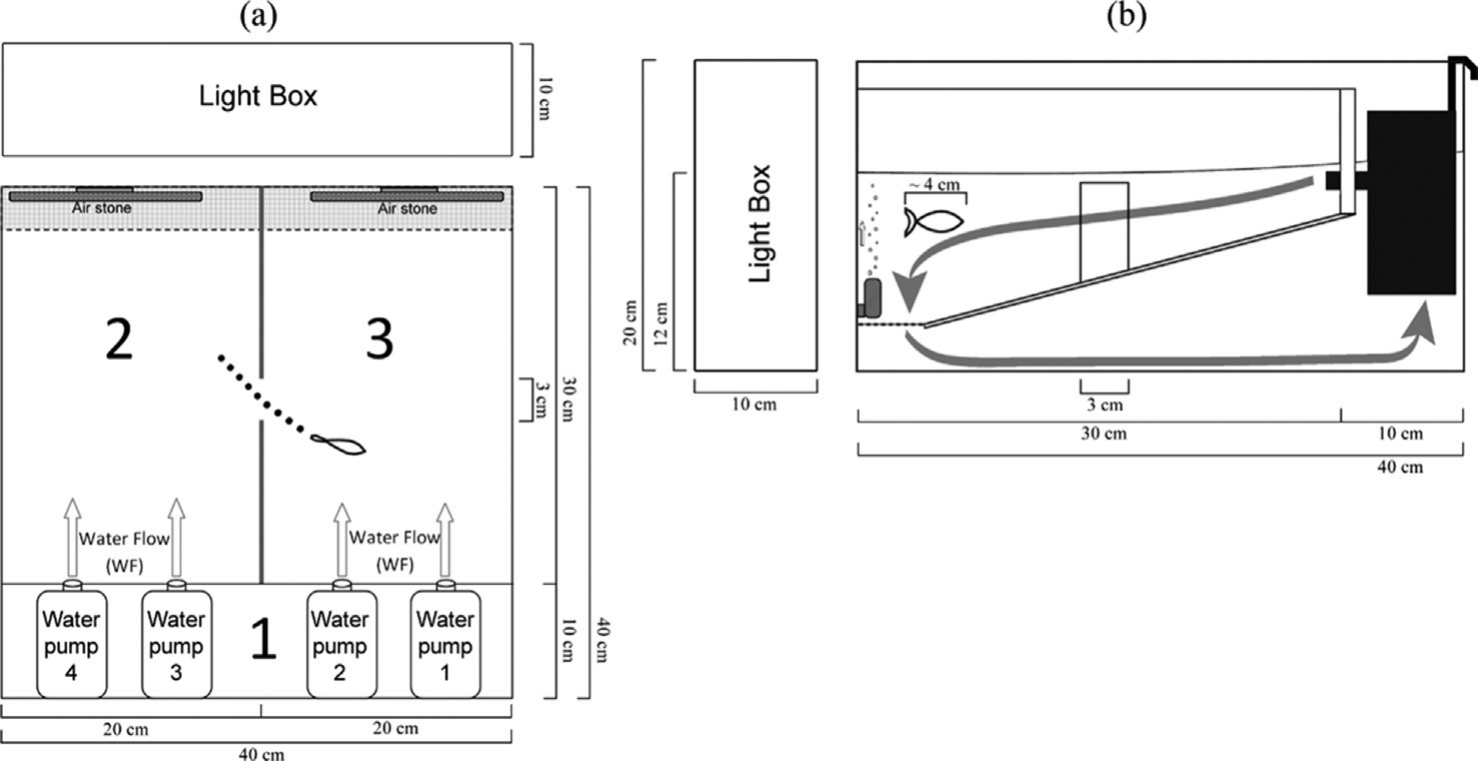
Overhead view (a) and side view (b) of the custom-made experimental shuttle tank, which was divided into 3 different compartments. Water pumps located in compartment 1 introduced WFs to the compartments 2 and 3. Compartment 1 was inaccessible to the fish. The fish could swim freely between compartments 2 and 3 through an opening that connected them. During signaled avoidance conditions, curtains of air bubbles (CABs) were introduced as warning stimulus using air stones attached to the back walls of compartments 2 and 3 (i.e., warning stimulus). A light box evenly illuminated the inside of the tank.

Fig. 1 provides an overview of the custom-made experimental tank filled to a depth of 12 cm with tap dechlorinated water (26°–27 °C) and divided into three compartments. Compartment 1 contained four water pumps that produced the water flows (WFs). This area was inaccessible to the subject. Fish could freely move across compartments 2 and 3 through a small opening (3-cm wide) that connected them. Mechanical issues with one of the four water pumps obligated to use only pumps 1 and 4 during the first sessions of the first experimental condition (see *Design* below). Immediately after the pump was repaired, all four pumps became again active during the subsequent sessions. However, inspection of the first treatment data obligated to use only pumps 1 and 4 during the rest of the treatment conditions.

Curtains of air bubbles (CABs) were introduced as warning stimulus during the signaled avoidance conditions (see design below) using two air stones attached to the back walls of compartment 2 and 3.

A custom-made system [2] was used to trace the fish in real time throughout each session. This system provided the location of the fish every 1/10th of a second. This output was processed in real time by another custom-made software that recorded the number of crossings between compartments and the amount of time that the subject was exposed to the WFs in each compartment. This software also controlled the timing of WFs and curtains of air bubbles (CABs – warning stimulus).

Each WF was scheduled to last 10s; any crossing that occurred during that period was automatically scored by the system as an escape response (i.e., interrupted the WF). Flow-Flow (F–F) and Response-Flow (R–F) intervals were implemented; both with a value of 30 s. Every crossing during the F–F and R–F intervals reset the 30-s period (see more details in Procedure).

### 2.3 Procedure

Each subject was individually exposed to daily 20-min sessions (including WF periods). The following are the details of each session of each condition of the experiment.

#### 2.3.1 Baseline

20-min sessions in which WFs were not delivered. The number of crossings between compartments and the amount of time spent in each compartment were recorded.

#### 2.3.2 Unsignaled avoidance (UA)

WFs were delivered every 30 s (F–F interval = 30 s) in the compartment in which the fish was located. If the subject crossed to the opposite compartment the WFs were immediately interrupted (escape) or postponed (avoidance). With each crossing the timer was reset to 30 s (R–F interval = 30 s). Additional to the abovementioned measurements conducted during baseline, on each UA session three more variables were recorded: (a) number and percentage of WFs delivered (percentages were calculated assuming that a total absence of crossings would have resulted in the delivery of 30 WFs – i.e., 100%); (b) duration and percentage of exposure to WFs (percentages were calculated assuming that a total absence of crossings would have resulted in a total exposure to WFs of 300 s – i.e., 100%); and (c) frequencies of different types of crossings, as described below.

*Escape* (Esc) responses were automatically scored if a crossing occurred during a WF. This response immediately interrupted the WF, and initiated an R–F interval (30 s).

*Avoidance* responses were automatically scored when crossings occurred during the R–F or F–F intervals. They also initiated an R–F interval. These crossings were further differentiated into *Early Avoidance* (EA) if they occurred during the first 25 s of the R–F interval (i.e., fish changed compartment within 25 s of the last response); *Late Avoidance* (LA) if they occurred during the last 5 s of the R–F interval; and *Flow-Flow avoidance* (FF) if they occurred anytime during the F–F interval.

#### 2.3.3 Signaled avoidance (SA)

Contingencies for the delivery of the WFs were the same as in the UA condition (F–F, R–F intervals, and definitions for escape and avoidance responses). The only difference was the presentation of a warning stimulus (curtains of air bubbles- CABs) during the last 5 s of each R–F interval. If the fish changed compartments during the warning stimulus, CABs were immediately interrupted and a new R–F interval initiated.

### 2.4 Design

A single-case experimental design was used [6]. The sequence of baseline, UA and SA conditions to which each subject was exposed is detailed in Table 1. Subjects changed to the next programmed condition when a steady pattern of crossing responses was observed across sessions (criteria based on [7], [8], [9]). Fish C01, C02, and C03 were initially exposed to UA prior to experiencing SA. Fish C04, C05, and C06 were exposed to the opposite sequence.

**Table 1:** Sequence of conditions for each subject and number of sessions per condition (between brackets).

Fish	Conditions									
C01	BL-1 (15)	UA-1 (22)	BL-2 (6)	UA-2 (6)	BL-3 (8)	SA-1 (13)	BL-4 (5)	SA-2 (5)		
C02	BL-1 (11)	UA-1* (16)	BL-2 (7)	UA-2 (14)	BL-3 (5)	UA-3 (5)	BL-4 (9)	SA-1 (6)	BL-5 (3)	
C03	BL-1 (11)	UA-1* (17)	BL-2 (7)	UA-2 (9)	BL-3 (5)	UA-3 (7)	BL-4 (5)	SA-1 (8)	BL-5 (6)	SA-2 (12)
C04	BL-1 (12)	SA-1* (16)	BL-2 (14)	SA-2 (10)	BL-3 (5)	SA-3 (8)	BL-4 (5)	UA-1 (11)		
C05	BL-1 (12)	SA-1* (16)	BL-2 (6)	SA-2 (9)	BL-3 (9)	SA-3 (6)	BL-4 (5)	UA-1 (9)	BL-5 (5)	UA-2 (12)
C06	BL-1 (15)	SA-1 (23)	BL-2 (5)	SA-2 (6)	BL-3 (8)	SA-3 (6)	BL-4 (5)	UA-1 (9)	BL-5 (5)	UA-2 (7)

Note. BL = Baseline; UA = Unsignaled Avoidance; SA = Signaled Avoidance; * = condition with two functional water pumps per compartment.

During the first treatment of subjects C02, C03, C04, and C05, WFs were produced by the two water pumps located on each compartment (in Table 1 marked with a “*”). Preliminary data analyses showed that, contrary to expected, the higher intensity of the WFs produced by the two pumps per compartment, as compared to one, resulted in a very low number of escape responses by the fish. This finding obligated to use a single water pump per compartment during the rest of the experiment. Fish C01, C02, and C04 were removed from the experiment before completing all the programmed conditions because of health issues.

### 2.5 Data analysis

Data of each individual was visually analyzed using guidelines for the type of experimental design implemented. For instance, considering differences in level and variations in trend across baseline and treatment conditions [10], [11].
